# Quality control of oceanographic in situ data from Argo floats using climatological convex hulls

**DOI:** 10.1016/j.mex.2017.11.007

**Published:** 2017-11-14

**Authors:** T.V.S. Udaya Bhaskar, R. Venkat Shesu, Timothy P. Boyer, E. Pattabhi Rama Rao

**Affiliations:** a Indian National Centre for Ocean Information Services (INCOIS), MoES, Hyderabad, India; b National Centers for Environmental Information (NCEI), Silver Spring, MD 20910-3282, USA

**Keywords:** Climatological convex hulls for outlier detection, Convex hulls, Point in polygon, Outliers, In situ data, Argo floats, classification

## Abstract

A new method of identifying anomalous oceanic temperature and salinity (T/S) data from Argo profiling floats is proposed. The proposed method uses World Ocean Database 2013 climatology to classify good against anomalous data by using convex hulls. An n-sided polygon (convex hull) with least area encompassing all the climatological points is constructed using Jarvis March algorithm. Subsequently Points In Polygon (PIP) principle implemented using ray casting algorithm is used to classify the T/S data as within or without acceptable bounds. It is observed that various types of anomalies associated with the oceanographic data viz., spikes, bias, sensor drifts etc can be identified using this method. Though demonstrated for Argo data it can be applied to any oceanographic data.

• The patterns of variation of the parameter (temperature or salinity) corresponding to a particular depth, along the longitude or latitude can be used to build convex hulls.
• This method can be effectively used for quality control by building Convex hulls for various observed depths corresponding to biogeochemical data which are sparsely observed.
• This method has the advantage of treating the bulk of oceanographic in situ data in a single iteration which filters out anomalous data.

## Graphical abstract

**Figure:**
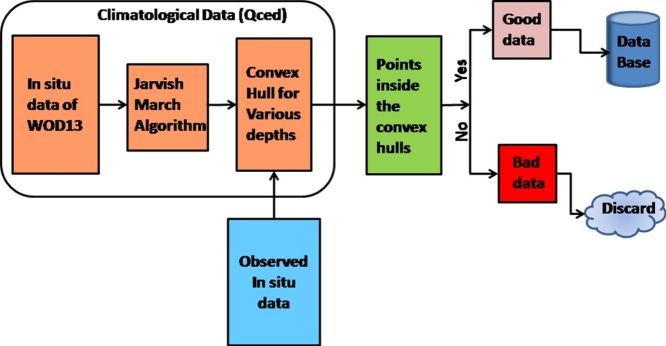

## Method details

### Background

Argo is an international program which has deployed more than 3000 autonomous floats in the global ocean. The Argo program is a major element of the Global Ocean Observing System (GOOS) and strives to observe the ever changing temperature and salinity fields of the upper ocean. In November 2007, the global Argo array of profiling floats has reached its initial target of 3000 operating floats worldwide. As of September 20, 2017, there are 3781 active floats worldwide. A defining aspect of Argo is that all data are reported in near real-time to meteorological forecasting centres and to the two Argo Global Data Assembly Centers (GDACs, localized in the USA and France), from which the accumulated data are made freely available without limitation to all the users [1].

An outlier is an observation that is abnormal compared to its neighbors and lies at an abnormal distance from other values in a random sample from a population. Spatial outliers are objects with distinct features from their surrounding neighbors in space. Detection of spatial outliers helps reveal important and valuable information from large spatial data sets. In the field of oceanography, for example, spatial outliers can be associated with natural events like cyclones, Indian Ocean Dipole, El Niño and the Southern Oscillation or can be associated with errors due to sensor malfunction/degradation. In oceanographic data, outliers are frequently represented in clusters, i.e., a group of observations from an instrument which is malfunctioning or from a float who's sensors have degraded over period after giving valuable data initially.

Many quality control procedures are prescribed for quality control of data from Argo floats. The ADMT had prescribed a set of 19 quality checks before the data is distributed to the users [2]. Further a secondary quality control methods based on scientific analysis called Delayed Mode Quality Control (DMQC) is also in place [3], [4]. But not all the data pertaining to global ocean are passed through DMQC. There are also some independent methods of quality control set up by some of the Data Acquisition Centres. Quality control method based on satellite altimetry was implemented by [5]. The methods of quality control of Argo and other in situ data archived at Coriolis Centre was described by [6]. A three way quality control method to qualify the profile data archived at INCOIS was proposed by [7]. However there are pros and cons with each of these methods.

**Fig. 1:**
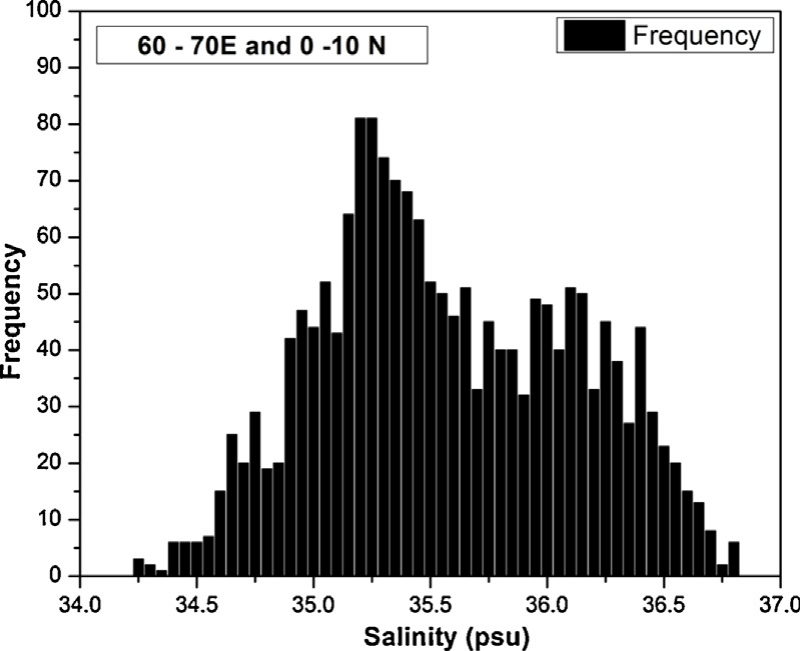
Frequency distribution of surface salinity in Arabian Sea. The data pertains to the 10° X 10° box of 60°–70° E and 0°–10° N.

Most of these methods work with a basic assumption that the data under consideration is normally distributed. These methods fails when the data are not normally distributed. If there exits multiple modes within the data, then the standard deviation check applied to the data may result in skewed output. As an example, the Arabian sea is divided into 10° X 10° boxes and a distribution plot of the surface salinity data is generated. It is observed that the data in the box 60°–70° E and 0°–10° N are bi-modally distributed (Fig. 1) owing to the formation and spreading of Arabian Sea High salinity water during winter in to the box mentioned above. In this case the mean and standard deviation will either be skewed to whichever of the two modes is most heavily sampled or the standard deviation would be very large with a mean somewhere in the middle of the two modes. In either case, quality control would be compromised. Similar observations near Gulf Stream led the World Ocean Atlas (WOA13) team to come up with updated version of World Ocean Atlas 2013 (refer Fig. 7 of woa12v2_changes.pdf). This paper describes a new method to augment existing procedures, so that the quality of the T/S profiles from Argo floats can be improved.

## Building the convex hulls for outlier detection

Argo data pertaining to the Indian Ocean are downloaded from the www.incois.gov.in web site. These data sets have been passed through the real-time quality control procedures detailed by the ADMT to be implemented by each Data Acquisition Center. All eligible profiles are passed through delayed mode quality control [2]. To test the proposed method, real time quality controlled profiles were considered.

**Fig. 2:**
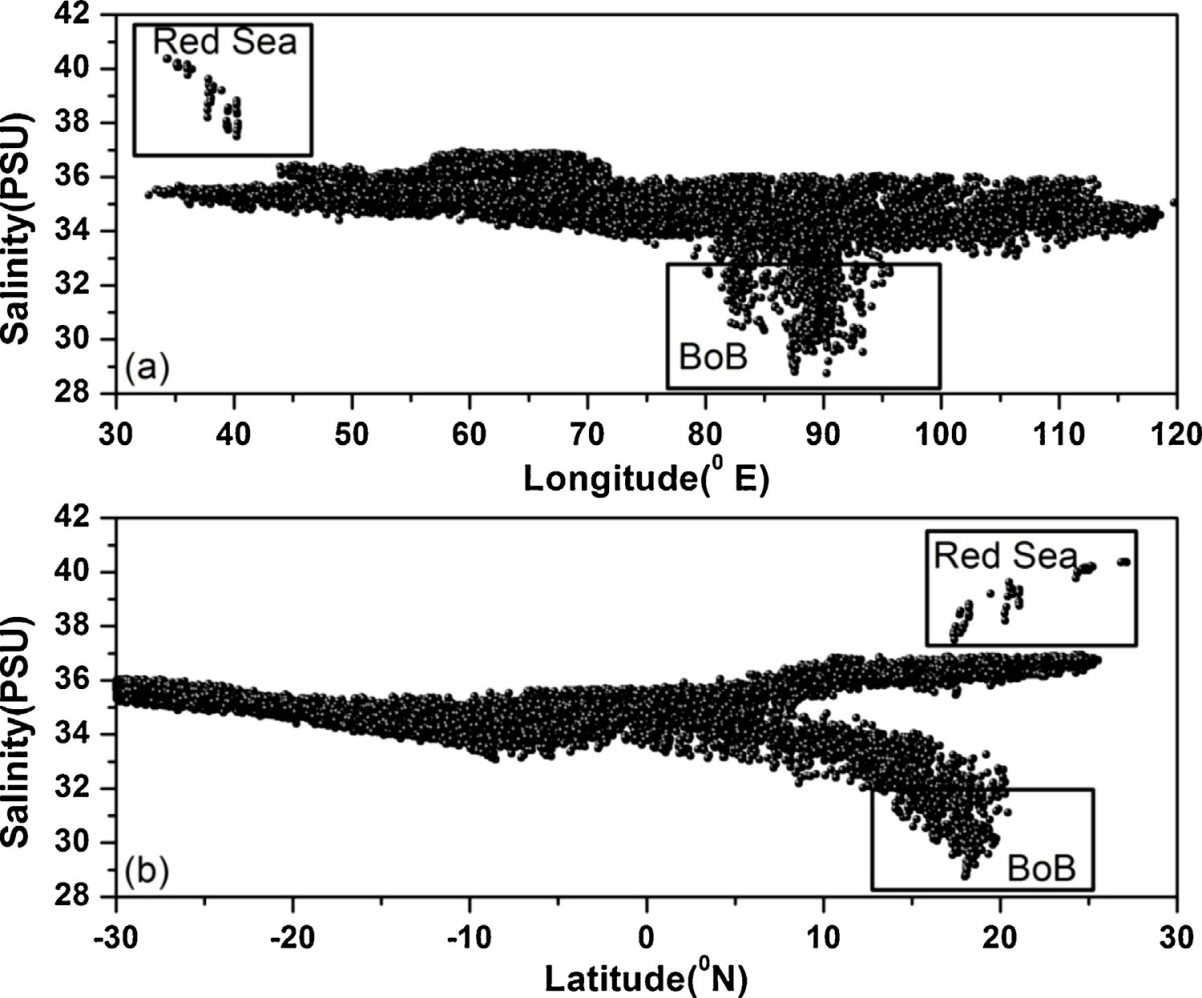
Patterns of salinity in the Indian Ocean when plotted against (a) longitude and (b) latitude corresponding to the depth 0 m obtained from World Ocean Database 2013.

The proposed method is based on the observation that temperature and salinity when plotted against longitude and latitude represent a certain pattern of the parameter variability for that region. Fig. 2 shows the salinity patterns for the Indian Ocean when plotted against the longitude (Fig. 2a) and latitude (Fig. 2b) corresponding to the 0 m depths obtained from the World Ocean Database 2013. From the patterns one can clearly demarcate different types of waters viz., the Red Sea and Bay of Bengal. Using this patterns a n-sided polygon is derived which is used for performing outlier analysis.

In the proposed method the principle of convex hull and Point-In-Polygon (PIP) together are used to identify anomalous Argo T/S profiles for a specified depth. The steps for application of the proposed method are as follows:

1. Observed Argo temperature and salinity profiles are interpolated using Akima spline [8] to the Levitus standard depths [9].
2. Using the World Ocean Database 2013 [10], [11] temperature and salinity data corresponding to each standard depth, an n-sided polygon (convex hull) is constructed with the least area encompassing the temperature and salinity fields with vertices of (latitude, temperature/salinity), (longitude, temperature/salinity). A Sample polygon (convex hull) for the 0 m depth of salinity is shown in Fig. 3.

**Fig. 3:**
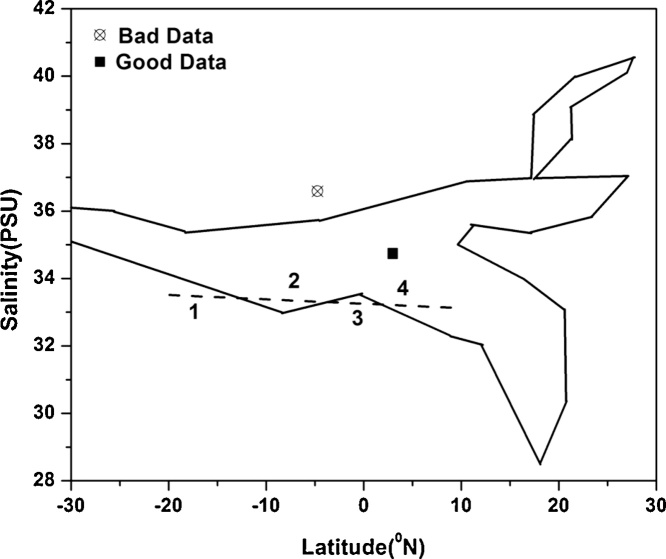
Generation of n-sided polygon (convex hull) based on the quality controlled climatological World Ocean Database 2013 and classification of good (filled square) and bad (crossed circle) data based on PIP and Jordan Curve Theorem. The number of intersections (numbered as 1–4 in figure) for a ray passing from the exterior of the polygon to any point; if odd, it shows that the point lies inside the polygon. If it is even, the point lies outside the polygon [12].
3. Subsequently the Point-In-Polygon (PIP) algorithm is used to check if the observed Argo temperature and salinity data (obtained in step 1) falls within or outside the n-sided polygon.
4. The quality flags of good(anomalous) data falling within(outside) the polygon are set, there by identifying wrong profile data (See Fig. 3).
5. Using the polygon (convex hull) corresponding to the deepest depth (∼2000 m) a check is made to discern if the Argo float sensors have any degradation etc.

It is worth mentioning that the method proposed here is applicable to two dimensions, however this can be extended to higher dimensions as well, where in a m-dimensional n-sided polyhedrons of latitude/longitude and temperature/salinity can be built and the observed profiles from Argo floats can be checked for outliers. However in this work we restrict the application of the proposed method to two dimensions only. The quality controlled climatology data used for the proposed method are temperature and salinity from World Ocean Database 2013 of the US National Centers for Environmental Information (NCEI). These quality controlled data are used to build polygons (convex hulls) for each standard depth. Since T/S profiles data from Argo floats are not available at regular depths, they are first uniformly interpolated to the standard depths represented in [9] using Akima spline [8]. The scattered observations thus obtained from Argo floats are checked to see if they are falling within the corresponding polygon (obtained from climatological data for that standard depth) using PIP algorithm. Jarvis March [13] algorithm also famously called as gift wrapping algorithm was used for constructing polygon (convex hull). This method is based on the principle of building a convex hull given a set of points and has a complexity of O(nh) where n is the number of points and h is the number of points on the convex hull. Fig. 3 shows a sample n-sided polygon (convex hull) built from WOD13 data at the ocean surface (0 m depth). Similar n-sided polygons for each standard depths are generated and used for qualifying the Argo temperature and salinity data. Once an n-sided polygon is constructed, the PIP algorithm is used to check if the observed Argo temperature and salinity data fall inside or outside the polygon.

## Point-in-polygon (PIP) implementation

In computational geometry, the PIP problem asks whether a given point in the plane lies inside, outside, or on the boundary of a polygon. It is a special case of point location problems and finds applications in areas that deal with processing geometrical data, such as computer graphics, computer vision, geographical information systems (GIS), motion planning, and Computer Aided Design.

There are many algorithms available to check whether the given point lies inside the polygon or not, like Crossing Test [14], Angle summation test, Triangle test [15] and Ray Casting Algorithm. In the present work the “Ray Casting Algorithm” was chosen for the purpose of identifying whether a given point lies inside or outside the algorithm. Fig. 3 shows a sample test for identifying whether a point is inside or outside the polygon. The Ray Casting Algorithm checks how many times a ray, starting from the point and going in ANY fixed direction, intersects the edges of the polygon. The number of intersections is an even number if the point is outside, and it is odd if inside. This algorithm is also known as the crossing number algorithm or the even-odd rule algorithm, and was known as early as 1962 [14].

The algorithm is based on a simple observation that if a point moves along a ray from infinity to the probe point and if it crosses the boundary of a polygon, possibly several times, then it alternately goes from the outside to inside, then from the inside to the outside, etc. As a result, after every two “border crossings” the moving point goes outside. This observation may be mathematically proved using the Jordan curve theorem [16]. In topology, a Jordan curve (simple closed curve) is a non-self-intersecting continuous loop in the plane. The Jordan curve theorem asserts that every Jordan curve divides the plane into an “interior” region bounded by the curve and an “exterior” region containing all of the nearby and far away exterior points, so that any continuous path connecting a point of one region to a point of the other intersects with that loop somewhere. Fig. 3 explains a sample scenario of how to determine whether a given data is good (bad) by virtue of it lying within (outside) the n-sided polygon. The biggest advantage of this proposed method is that large number of profiles data can be checked for their quality without manual intervention.

## Validation of the proposed method

In general temperature and pressure sensors are found to be robust and salinity sensors on Argo floats are susceptible to changes, degradation owing to bio-fouling [3]. Some of the recorded problems with salinity sensors are, offsets, freshening due to Tri-Butyl Tin Oxide (TBTO), drift after a set of cycles etc. Hence the importance in checking the quality of salinity data. Here we will check the Indian Ocean Argo salinity data using the proposed method. Quality controlled climatological data of salinity corresponding to the profiling depth of the floats under consideration are obtained from WOD13. An n-sided polygon (convex hull) is constructed using Jarvis March algorithm. Argo float time series for the profiling depth values are then obtained and checked against this n-sided polygon using PIP algorithm. If the points fall outside the polygon, the Argo time series is suspected to have a problem (drift, bias, spike etc) or represent anomalous oceanic condition. Because the climatology incorporates a large number of observations spanning decades, a float is suspected to have a problem if the profiling depth salinity points fall outside the n-sided polygon.

**Fig. 4:**
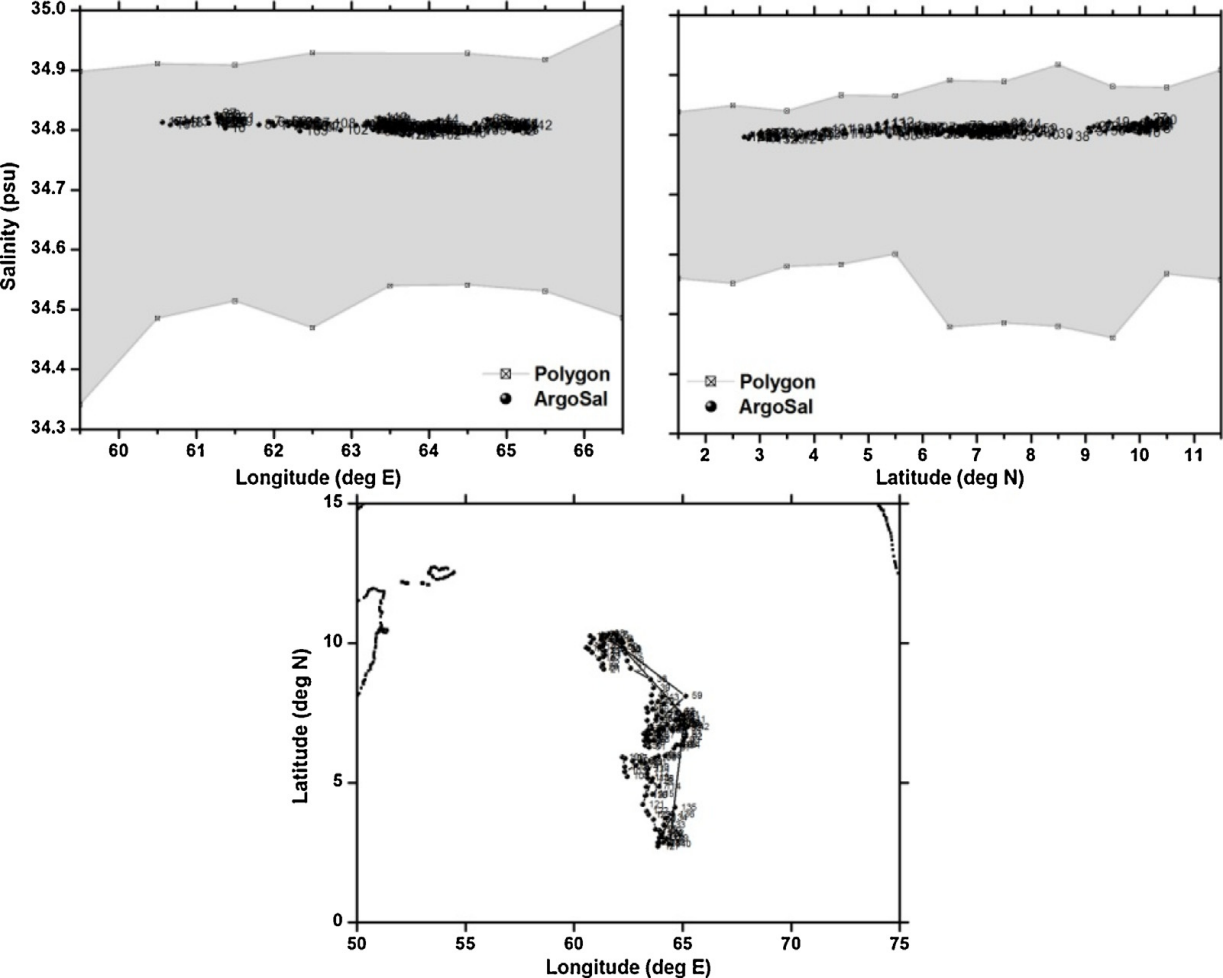
(a) n-sided polygon (grey shaded region) constructed with longitude, salinity observations corresponding to the profiling depth (2000 m) of float WMOID 2900782. (b) same as (a) but with latitude. (c) Trajectory of the float during its life time.

To demonstrate the robustness of the proposed method, 5 typical floats are chosen which represent different problems like drift, offset, grey listed etc. The details of the floats chosen for the validation are given in Table 1. These examples include good and anomalous floats, together with their positions and n-sided polygons for their respective profiling depth. The first float, identified as WMO 2900782 travelling southward in the Arabian Sea, is a typical example of a good float. All the profiles are observed to be good with the salinities corresponding to the profiling depth falling within the n-sided polygon built from the WOD13 climatological data (Fig. 4).

**Table 1:** Details of the floats chosen for validation of polygon method.

S.No	WMOID	First Cycle	Last Cycle	Total Cycles	Parking/Profiling depth	Type of problem
1.	2900782	22/06/2007	07/01/2012	167	2000/2000	Good
2.	2900877	11/09/2007	04/09/2012	183	2000/2000	TBTO Fouling
3.	2901340	25/12/2011	19/12/2014	110	2000/2000	Freshening for short duration
4.	2900783	09/07/2007	29/12/2012	201	2000/2000	Grey Listed float
5.	2900554	06/09/2005	27/06/2009	279	1000/2000	Salinity drift

**Fig. 5:**
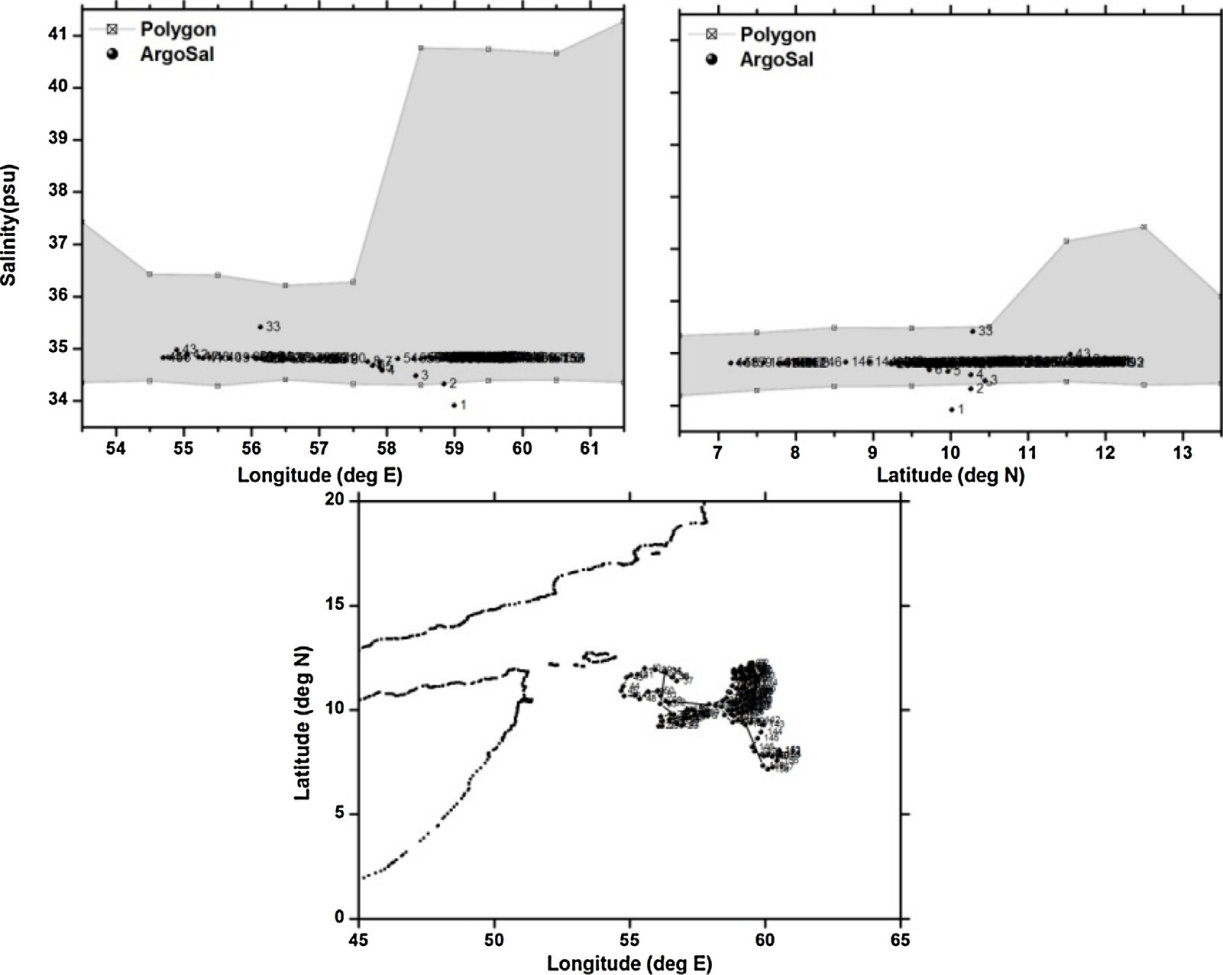
(a) n-sided polygon (grey shaded region) constructed with longitude, salinity observations corresponding to the profiling depth (2000 m) of float WMOID 2900877. (b) same as (a) but with latitude. (c) Trajectory of the float during its life time.

The second example (WMO 2900877) is contaminated by TBTO fouling which is evident from the initial fresher salinity profiles. The conductivity cell drifts because of the possible change in the dimension of the conductivity cell due to fouling. TBTO is used to improve the anti-fouling in the conductivity cell [17]. Sometime this causes erroneous freshening in the initial profiles until the coating is washed off. Clearly one can see all the initial profiles falling outside the n-sided polygon (Fig. 5), thereby indicating the case of the TBTO contamination. Also one can see the few profiles (cycle 33, 43) which are spikes in the time series of the float which are observed to fall outside the polygon.

**Fig. 6:**
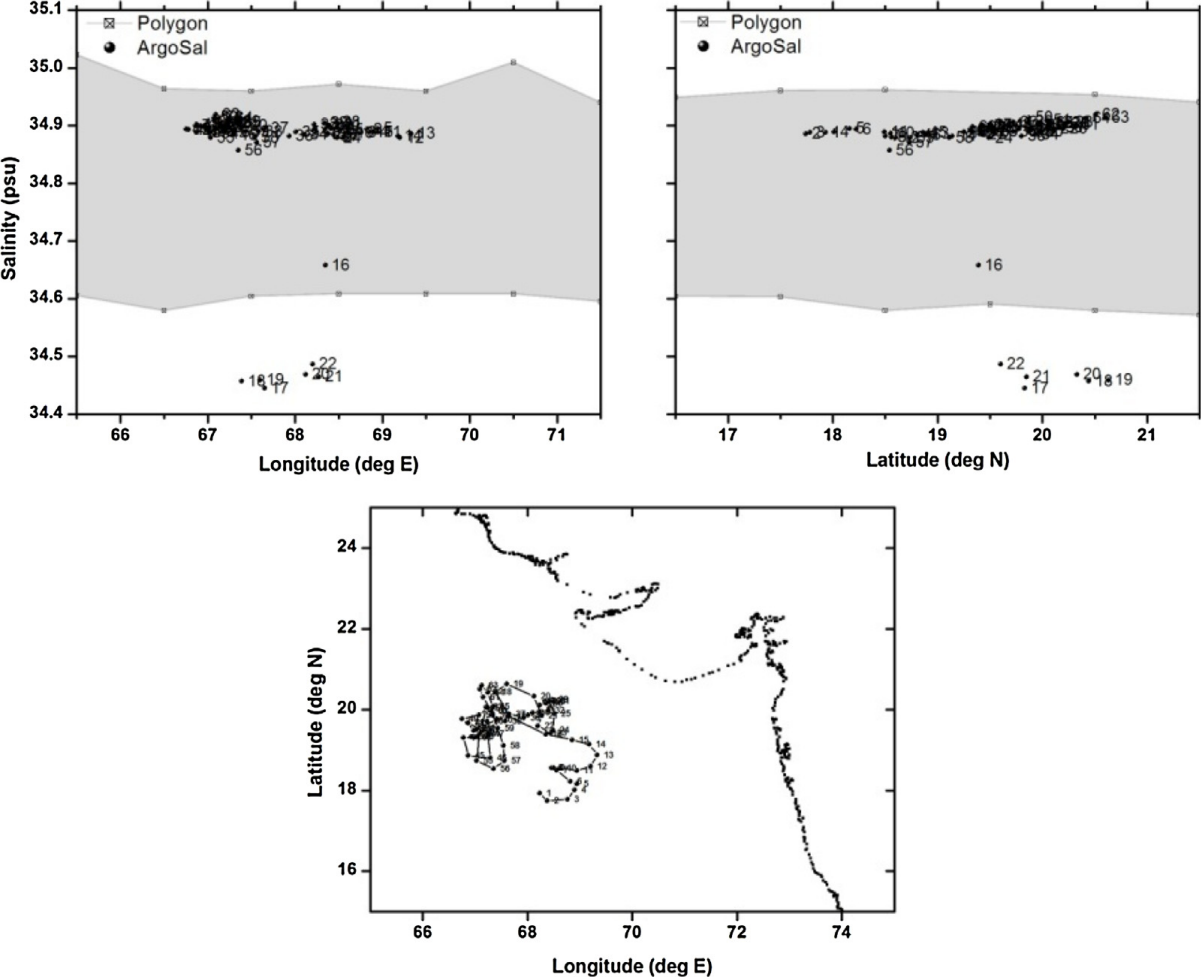
(a) n-sided polygon (grey shaded region) constructed with longitude, salinity observations corresponding to the profiling depth (2000 m) of float WMOID 2901340. (b) same as (a) but with latitude. (c) Trajectory of the float during its life time.

**Fig. 7:**
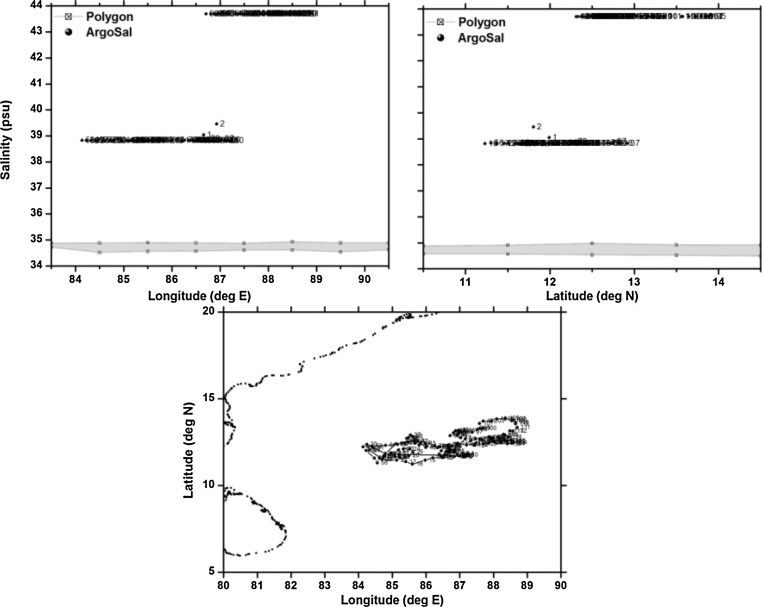
(a) n-sided polygon (grey shaded region) constructed with longitude, salinity observations corresponding to the profiling depth (2000 m) of float WMOID 2900783. (b) same as (a) but with latitude. (c) Trajectory of the float during its life time.

The third example is a float which is observed to have drift in the salinity sensor only for few cycles. This float is observed to have salinity drift between cycles 16–22. This can sometime happen due to some biological matter entering into the conductivity pipe. When the biological matter is washed out, the salinity sensor tends to come back to normalcy. This is clearly observed by the corresponding salinity values at profiling depth of 2000 m falling outside the n-sided polygon (Fig. 6). This floats seems to have recovered to normalcy after cycle 22. The float with WMO 2900783 in the Bay of Bengal (Fig. 7 shows a typical case of a float whose salinity at profiling depth (2000 m) is completely offset to that of the climatology. All the salinity values corresponding to this float are observed to be falling well outside the n-sided polygon right from the cycle 1. This is typical case of a greylisted float. Greylisting is used for real-time operations of Argo floats, to detect a sensor malfunction. It is a list of suspicious or malfunctioning float sensors and is managed by each Data Acquisition Centre (DAC).

**Fig. 8:**
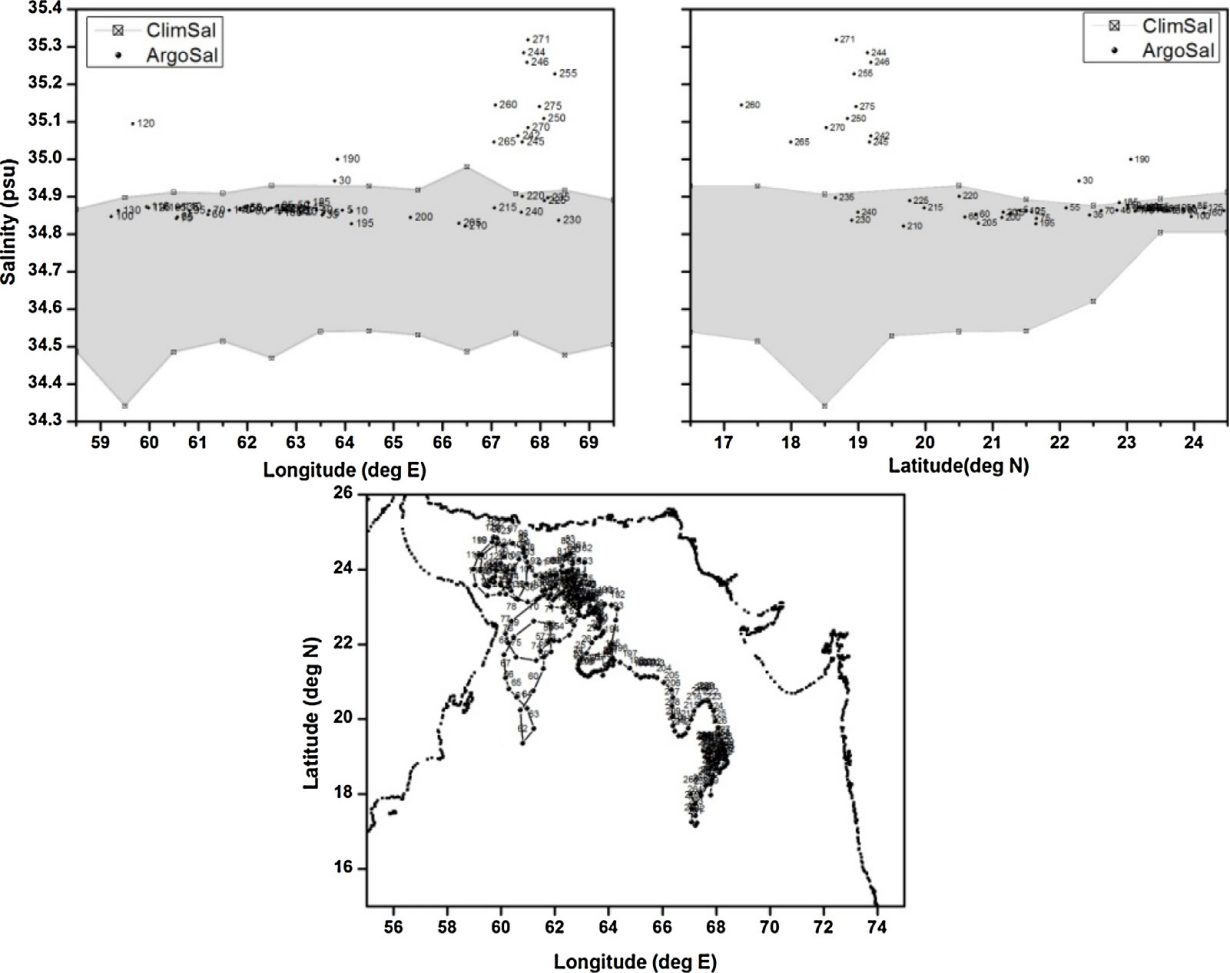
(a) n-sided polygon (grey shaded region) constructed with longitude, salinity observations corresponding to the profiling depth (2000 m) of float WMOID 2900554. (b) same as (a) but with latitude. (c) Trajectory of the float during its life time.

The last example is that of a float with WMO 2900554 in the Arabian Sea (Fig. 8) whose salinity started to drift starting from cycle 200. All the salinity values fall outside the n-sided polygon from this cycle onwards. The examples discussed above are only an illustration of the possible cases among which the anomalous floats fall including spikes, offsets, and drift. These examples demonstrate the usefulness of the proposed method for identifying bad profiles. The biggest advantage of the proposed method is its applicability to suite of float data in a single test which can easily detect good against anomalous profiles. For better results this methods can be augmented with other methods in use by the Argo community like altimetry based QC and objectively analyzed based QC.

## Additional information

The number of profiles obtained annually by Argo floats in the world oceans was more than 30,000 in 2003 and this number increased to about 173,524 profiles in 2016. There are around 84,076 CTD casts in World Ocean Database (WOD) which reach deeper than 2000 m and in total there are 115,808 CTD casts in WOD deeper than 1500 m which makes Argo’s recent yearly contribution greater than all historic CTD casts deeper than 1500 m. Argo in conjunction with many other data sets are used in studies related to climate change, sea level rise, ocean heat content, mixed layer processes, assimilation into ocean models (GODAS), generation of better analysis products etc. However some of the studies require that the data from these instruments be of high quality as the results might be sensitive to biases or instrument errors. These anomalous data are to be identified and eliminated or flagged before the data is put to use.

Over the past decade or more, geographic distribution of oceanographic T/S profile data has become more uniform owing to the deployment of Argo profiling floats. From running the ocean models operationally to the preparation of climatologies, oceanographic data is widely put to use. Argo floats deployed by various countries are deployed by different groups who use different types of pressure and CTD sensors. Each country has their own choice of setting the measurement resolution for obtaining the T/S samples. Owing to different methods of measurements, different instruments and differences in handling the data, there is the possibility of leaving some anomalous data unnoticed. Even though the Argo Data Management Team (ADMT) has implemented a system of quality checks [2], as different organizations/institutions employ additional methods for performing quality checks on data, there can be scope for existence of erroneous data. It would be a cumbersome process to individually pin point these anomalous data even though various methods of handling these datasets are developed.

## References

[1] Riser S.C. (2016). Fifteen years of ocean observations with the global Argo array. Nat. Clim. Change.

[2] A.P.S. Wong, Robert Keely, Thierry Carval, and the Argo Data Management Team, Argo quality control manual, ver 3.0, 2016, 56 pp.

[3] Wong A.P.S., Johnson G.C., Owens W.B. (2003). Delayed-mode calibration of autonomous CTD profiling float salinity data by theta-S climatology. J. Atmos. Ocean. Technol..

[4] Owens W.B., Wong A. (2009). An improved calibration method for the drift of the conductivity sensor on autonomous CTD profiling floats by y–S climatology. Deep-Sea Res. I.

[5] Guinehut Stephanie, Coatanoan Christine, Dhomps Anne-Lise, Le Traon Pierre-Yves, Larnicol Gilles (2009). On the use of satellite altimeter data in argo quality control. J. Atmos. Ocean. Technol..

[6] Cabanes C., Grouazel A., von Schuckmann K., Hamon M., Turpin V., Coatanoan C., Paris F., Guinehut S., Boone C., Ferry N., de Boyer Montégut C., Carval T., Reverdin G., Pouliquen S., Le Traon P.Y. (2013). The CORA dataset: validation and diagnostics of in-situ ocean temperature and salinity measurements. Ocean Sci..

[7] Udaya Bhaskar T.V.S., Pattabhi Rama Rao E., Venkat Sheshu R., Devender R.D. (2012). A note on three way quality control of argo temperature and salinity profiles – a semi-automated approach at INCOIS. Int. J. Earth Sci. Eng..

[8] Akima H. (1970). A new method of interpolation and smooth curve fitting based on local procedures. J. Assoc. Comp. Mac..

[9] Levitus S., Burgett R., Boyer T.P. (1994). World Ocean Atlas 1994 Volume 3: Salinity. NOAA Atlas NESDIS 3.

[10] Locarnini R.A., Mishonov A.V., Antonov J.I., Boyer T.P., Garcia H.E., Baranova O.K., Zweng M.M., Paver C.R., Reagan J.R., Johnson D.R., Hamilton M., Seidov D., Levitus S. (2013). World Ocean Atlas 2013, Volume 1: Temperature.

[11] Zweng M.M., Reagan J.R., Antonov J.I., Locarnini R.A., Mishonov A.V., Boyer T.P., Garcia H.E., Baranova O.K., Johnson D.R., D.Seidov M.M., Levitus S. (2013). World Ocean Atlas 2013, Volume 2: Salinit.

[12] Huang Chong-Wei, Grouazel Shih (1997). On the complexity of point-in-polygon algorithms. Comput. Geosci..

[13] Jarvis R.A. (1973). On the identification of the convex hull of a finite set of points in the plane. Inform. Process. Lett..

[14] Shimrat M. (1962). Algorithm 112, Position of Point Relative to Polygon.

[15] Badouel Didier, Glassner Andrew S. (1990). An Efficient Ray-Polygon Intersection, Graphics Gems.

[16] Jordan C. (1893). Cours D’Analyse De l’ Ecole Polytechnique, Paris.

[17] Thadathil P., Muraleedharan P.M., Gopalakrishna V.V., Reddy G.V., Ratnakaran L., Revichandran C., Murthy V.S.N. (2004). Validation of ARGO data in the indian ocean. Gayana.

